# "No pain, no gain": Simulation-based learning in teacher education: The mediating role of simulation hindrances

**DOI:** 10.1371/journal.pone.0317255

**Published:** 2025-01-13

**Authors:** Orna Levin, Rivi Frei-Landau, Chen Goldberg

**Affiliations:** 1 Department of Education & Leadership, Achva Academic College, Shikmim, Israel; 2 Department of Special Education, Achva Academic College, Shikmim, Israel; 3 The Open University, Ramat-Gan, Israel; 4 University of Haifa, Israel; 5 Gordon College of Education, Haifa, Israel; Murcia University, Spain, SPAIN

## Abstract

Research on educational simulations has attempted to decipher the simulation-based learning (SBL) process by examining factors that facilitate and impede this process. In the current study, we examined the role of SBL participants’ hindrances, in particular their experience of anxiety or fear, which we view using the framework of leaving one’s comfort zone. Departure from one’s comfort zone has never been studied in the context of SBL in teacher education. A quantitative analysis of data collected via a questionnaire on Simulation Learning Outcomes in Teacher Education (SLOTE) revealed a model in which the hindrance variable potentially mediated all of the relationships between the background variables and the simulation learning outcomes (i.e., communication skills and collaborative learning insights). These results improve the theoretical understanding of the SBL process in this field and indicate ways to optimally utilize potential hindrances to plan and apply SBL for the purpose of learning.

## Introduction

Simulation-based learning (SBL) is a mediation tool that simulates the professional arena for the purpose of learning [1]. The simulation technique was initiated in the fields of military and medical training [2–4], whereas the use of SBL in the field of teacher education is in its early stages [5–7]. In an effort to understand the mechanism underlying learning through simulation so as to design better educational simulations, recent studies have examined the factors that may facilitate or hinder the learning process during SBL [8, 9].

The focus of the current study was on factors that may potentially impede the learning process during simulations, which we refer to as "SBL hindrances". These may include an experience of embarrassment, stress, anxiety stemming from the personal exposure while participating in SBL. It is assumed that participating in a simulation entail leaving one’s "comfort zone" for learning purposes. This concept suggests that when people face a situation that requires them to leave their comfort zone, push the envelope, and stretch the limits of their performance, they will ultimately respond by overcoming the inconvenience and the associated stress or fear, which in turn enables them to learn and grow (i.e. learn) as individuals [10, 11]. This study is a first attempt to explore the concept of leaving one’s comfort zone in the context of learning through simulation in the field of teacher education.

### Theoretical background

#### Simulation-based learning

SBL is an active learning experience involving collaborative reflective group learning [12, 13]. The affordances of SBL, which have been comprehensively documented in the professional literature [7, 14], include the learning of skills needed to perform professionally in this field [15, 16]; the enhancement of social skills [17], and the practice of reflective thinking [18]. Nevertheless, some studies reported negative aspects too, such as cognitive overload [19], and the impact of the emotional demands of this learning technique, which have yet to be fully addressed, particularly in the field of teacher education [20]. As positive emotions help facilitate a person’s achievement of optimal best practices [21], it is worthwhile to deepen our understanding of learners’ negative experiences and their impact on SBL outcomes.

The clinical simulation model used in this study involved predefined scenarios that are enacted by a learner accompanied by a professional actor and video-recorded in real-time, while the other participants observe the performance of the scenario by their fellow learner interacting with a professional actor [22]. The content of the scenario is derived from learners’ real-life professional challenges [23, 24] After the simulation is performed, the participants engage in a reflective, peer-learning-based debriefing [25], guided by the simulation facilitator [26, 27]. As such, during both the simulation enactment and the debriefing, learners are liable to experience embarrassment and stress [28, 29], an aspect that requires further investigation regarding its potential impact on the learning process.

Overall, the clinical SBL workshop involves four consecutive generic stages: (1) An opening phase with a facilitator introducing the activities. (2) A five-minute "simulated experience" is enacted by a professional actor who interacts in real-time with a learner-volunteer. (3) The facilitator conducts a group-wide debriefing phase, during which the participants share and discuss the facts (“what happened here?”), the possible causes and motives behind the events (“why did this happen?”), and the lessons they derived from the experience (“what will we choose to do next time?”). (4) After the group debriefing process, the actor presents his/her feedback, while the volunteer and the rest of the group members listen. Thus, the workshop concludes with a group participants’ reflective insights. As appears, enacting the scenario and participating in the debriefing process can be a source of some degree of embarrassment or distress, as it exposes one authentic reactions and responses to others’ observations and critiques.

#### Learning through simulation entails leaving one’s comfort zone by experiencing hindrances

As explained, SBL entails leaving one’s comfort zone [30], whereby the extent of the stress caused by this departure depends on the efficacy of the learning process [31]. More than 100 years ago, Yerkes and Dodson [32] claimed that entering what they termed the “danger zone” should be done cautiously, because stress can enhance performance up to a point of optimal attentiveness; however, beyond this point, the performance deteriorates as the stress increases.

A comfort zone is a psychological state in which individuals feel secure, familiar, and in control, experiencing minimal stress or anxiety. This concept suggests that when individuals perform in familiar situations, they feel safe and tend to avoid challenges that may induce discomfort [10]. In contrast, the danger zone refers to a state in which the level of stress exceeds the optimal threshold for performance and learning, impairing cognitive and physical performance, which leads to a decline in effectiveness and hinders learning and task completion [32].

The comfort zone model is based on the belief that when people are asked to perform in a situation with which they are not familiar, they tend to experience stress, anxiety, and discomfort, all of which makes coping with the situation more challenging [10], whereby successful coping to learning and growth [33]. The role of the perceived danger is an integral part of the departure from the comfort zone [34]. Moreover, in this model, personal growth depends on the participant experiencing stress [11, 35]. Yet it should be noted that if the departure from the comfort zone is uncontrolled, which in the language of simulation means it takes place in and insecure environment, then the “stretch zone,” which is seen as promoting effective learning, can become a danger zone or even a panic zone, where no learning is possible [33].

In the research literature on clinical simulations in the field of medicine, the departure from one’s comfort zone is measured by the level of stress experienced by the participant. It is understood that the simulation experience leads to a wide range of emotions and feelings, including those that promote learning and others that impede learning. Chief among them, however, is the experience of stress [36], which is perceived as a characteristic of simulation practice [37].

In simulations conducted with preservice teachers, which focused on their emotional experiences, participants reported experiencing tension and stress [38]. Moreover, it was found that the stress that accompanied the experience was perceived as a positive factor that is characteristic of the simulation experience [36]. This type of stress is unique to SBL in contrast to traditional forms of learning [39].

Studies that examined the potential of stress to impede SBL found that the stress is experienced in waves that wax and wane [40]. Other studies found that too much stress could decrease the participant’s functioning and ability to apply the necessary skills [41, 42]. Bong et al. [39] compared the participants’ stress levels during the simulation with the learning outcomes after the simulation, claiming that it would be possible to lower the stress levels without compromising the effective learning achievements. This assumption was based on the perception that stress is a debilitating factor in the learning process and its role should be minimized. Their findings demonstrated that the stress experienced by those acting out the simulation was greater than that which was experienced by the observers [39]. Another study that examined the role of stress in SBL in the field of nursing highlighted the assumption that people’s stress threshold can differ significantly and so too their ability to cope with the environmental changes inherent in SBL [43]. They suggested that the potentially negative influence of stress in SBL is related to the participants’ exposure to unfamiliar training environments [44], and that stress has a direct effect on participants’ performance in the simulation training [45], in terms of both technical and substantial aspects of learning [46]. In the field of medicine, the most experienced extreme claim was that stress experienced in SBL could lead to the presentation of posttraumatic symptoms [47, 48].

At the other end of the spectrum, it was found that positive stress is necessary for optimal simulation performance [49, 50]. A review study that focused on the effects of stress on nursing students using SBL [36] found that in 17 studies, the participants reported medium levels or high levels of stress related to the simulation, yet at the same time they also rated the SBL experience as an extremely valuable tool. Moreover, a recent systematic review comprises of sixty-two studies in nursing education [51] revealed that simulation is an effective strategy for reducing anxiety and increasing self-confidence compared to conventional teaching strategies. Despite the stress-related concerns, it appears that stress is an immanent part of and figures prominently in the SBL process, wherein difficulties and mistakes are considered a valuable part of the learning experience [30].

In summary, stress levels represent an aspect which may impede the SBL experience. There has been little study of the factors that impede SBL in the field of teacher education, and especially, there is a paucity of research from the perspective of the model regarding the departure from one’s comfort zone. Thus, for example, in a recent review of 15 studies on SBL in teacher education, there was no mention of stress in any of the studies reviewed [7]. The current study aims to address this lacuna, in order to gain a more profound understanding of the role played by SBL hindrances which may impede learning through simulations.

### The study hypotheses

- The existence of factors that impede learning will be related to positive SBL outcomes (measured as communication skills and collaborative learning insights). Hence, participants’ report of higher levels of hindrances will correlate with their reports of higher rates of learning outcomes.
- The relationship between participants’ background characteristics (age, gender, and years of experience) and the learning outcomes will be mediated by their experience of simulation-related hindrances.
- Participants’ role as either actor or observer will be related to their experience of hindrances and to their learning outcomes.

## Methods

### The study context

The research was conducted at a simulation center located in XXX, which operates according to the clinical simulation model. Generally, the workshops’ focus is on promoting communication skills, teamwork, and multiculturalism. The center offers workshops to both preservice and in-service teachers in separate groups. Workshops are guided by a trained facilitator. Each simulation workshop lasts four hours and includes approximately 12–15 participants. Each workshop comprises various scenarios involving typical conflictual situations in the field of education (see [52] for a detailed description of the simulation scenarios).

### The study design

The current study is an extension and a deeper investigation into the data collected in a previous study conducted by the authors [8]. There, the authors presented a validated tool used to assess SBL outcomes in teacher education. The database and instruments used in the previous study are examined here with the intent of assessing the effect of potential hindrances on the relationship between the two dimensions of SBL outcomes (communication skills, collaborative learning insights) and the type of role played by the participants (i.e., participant as actor versus observer). This is a quantitative study of the data collected previously using the tools validated in the previous study. In this manner, we were able to examine additional aspects and relationships among the study variables, which were beyond the framework of the previous study.

### Participants

The study’s participants were 370 SBL participants who partook in SBL workshops at the simulation center, in the years of 2019–2020. More specifically, the recruitment period for this study was between May 12, 2019, to May 12, 2020. Among the 370 participants, 78 were preservice teachers (22%) and 292 were in-service teachers (78%). The vast majority of participants were women (92%). Participants’ age ranged from 22 to 67, with an average of 41.1, and their teaching experience ranged from 0 to 38-years, with an average of 14.4 years. Similarly, participants’ level of education ranged from 12 to 25 years, with an average of 17.4 years. Demographic data of the participants are presented in Table 1.

**Table 1:** Overview of participants’ background variables.

Background Variables		
		Sample
Participants’		370
Gender	% female	92.2
	% male	7.8
Age	Mean age (in years)	41.1
	Range age	22 to 67
Years of Schooling	Mean (in years)	17.4
	Range	12 to 25
Teaching Experience	Mean teaching experience (in years)	14.4
	Range teaching experience	0 to 38
	% preservice teachers	21.1
	% inservice teachers	78.9
Simulations	% participants as actors in current simulation	16.8
	% observer in current simulation	83.2

### Data collection instruments

#### Demographic questionnaires

Participants completed questionnaires denoting their gender, age, teaching experience, professional specialty, and role in the simulation workshop.

#### The Simulation-based Learning Outcomes in Teacher Education (SLOTE) scale

A questionnaire developed and validated in previous study [8]. This scale–the SLOTE scale–is a 29-item questionnaire to which participants responded using a Likert scale, ranging from not at all (1) to very much (7), indicating the extent to which, in their experience, the SBL workshop had resulted in a specific outcome (item). The 29 items are presented in Appendix 1. Overall, this scale consists of three factors: peer reflective learning (α = .86), simulation-related hindrances (α = .74), and communication skills (α = .95). Descriptive statistics and Pearson correlations of simulation outcomes and background variables are presented in Tables 2 and 3.

**Table 2:** Descriptive statistics of simulation outcomes and background variables.

		Gender			Teaching Experience			Role in Simulation		
		Female	Male	*t*	Inservice teachers	Preservice teachers	t	Observer	Participator	*t*
	N	341	29		292	78		308	62	
Communication Skills	M	5.7	5.2	1.953^^^	5.5	5.9	2.569*	5.6	5.8	.966
	SD	1.2	1.1		1.2	1.0		1.2	1.1	
Simulation-related Hindrances	M	4.1	3.6	2.173*	4.0	4.7	4.220**	4.1	4.0	.708
	SD	1.3	1.4		1.4	1.3		1.4	1.3	
Collaborative Learning Insights	M	6.1	5.8	1.601	6.0	6.2	1.584	6.0	6.3	2.014*
	SD	1.0	1.2		1.0	0.9		1.1	0.7	

**p < .01

*p < .05

^*p* < .06

**Table 3:** Pearson correlations–between background variables (age, education, and teaching experience) and simulation outcomes.

	Communication Skills	Collaborative learning Insights	Simulation-related Hindrances
Age	-.070	-.201**	-.053
Education (years)	-.055	-.156**	.021
Teaching Experience (years)	-.073	-.182**	-.071

** *p* < .01

*p < .05

### Procedure

The study was approved by the Institutional Ethics Committee. All participants were informed about the study and gave their informed consent in writing. As the instrument was administered via a link to a Google Form, participants could refuse to participate without experiencing any inconvenience. Furthermore, efforts were made to meticulously maintain the rights, privacy, anonymity, and confidentiality of the participants throughout the research process, by upholding professional ethical standards.

### Data analysis

We applied structural equation modeling (SEM) using the maximum likelihood (ML) method [53] to measure error and path coefficients. According to Steiger [54] and Kline [55], the appropriateness of a theoretical model for the data analyzed must be proven, based on the empirical findings. In this case, this was addressed by following several indices of ML measures: Goodness of fit index (GFI), comparative fitness index (CFI) [56], root mean square error of approximation (RMSEA), the *χ*^*2*^ statistic, and the *χ*^2^*/df* ratio. The model is considered appropriate for the data when the *χ*^2^*/df* <3 and is not significant. A model is also considered appropriate for the data when the CFI is higher than 0.95 and the RMSEA is lower than 0.05 [57]. The analysis was carried out using IBM AMOS version 27.

## Results

The SEM analysis revealed that the model was adequate and was thus compatible with our data (*χ*^2^ = 5.268, *df* = 8, *χ*^2^*/df* = 0.658, *p* = 0.729; NFI = 0.989; CFI = 1.000; RMSEA = 0.000) as presented in Table 4.

**Table 4:** Fit indices of the proposed research models testing the relationships between participants’ communication skills, collaborative learning insights, simulation-related hindrances, and background characteristics (*N* = 370).

Fit indices		Recommended level of fit	Value of the measure
Absolute fit	*χ* ^2^_(*df* = 8)_	*p* > 0.05	5.268, *p* = .729
	RMSEA	<0.08	.000
	PCLOSE	>0.05	.968
Incremental fit	CFI	>0.90	1.000
	TLI	>0.90	1.011
	NFI	>0.90	.989
Parsimonious fit	$\frac{\chi^{2}}{df}$	<3	.658

**Fig 1:**
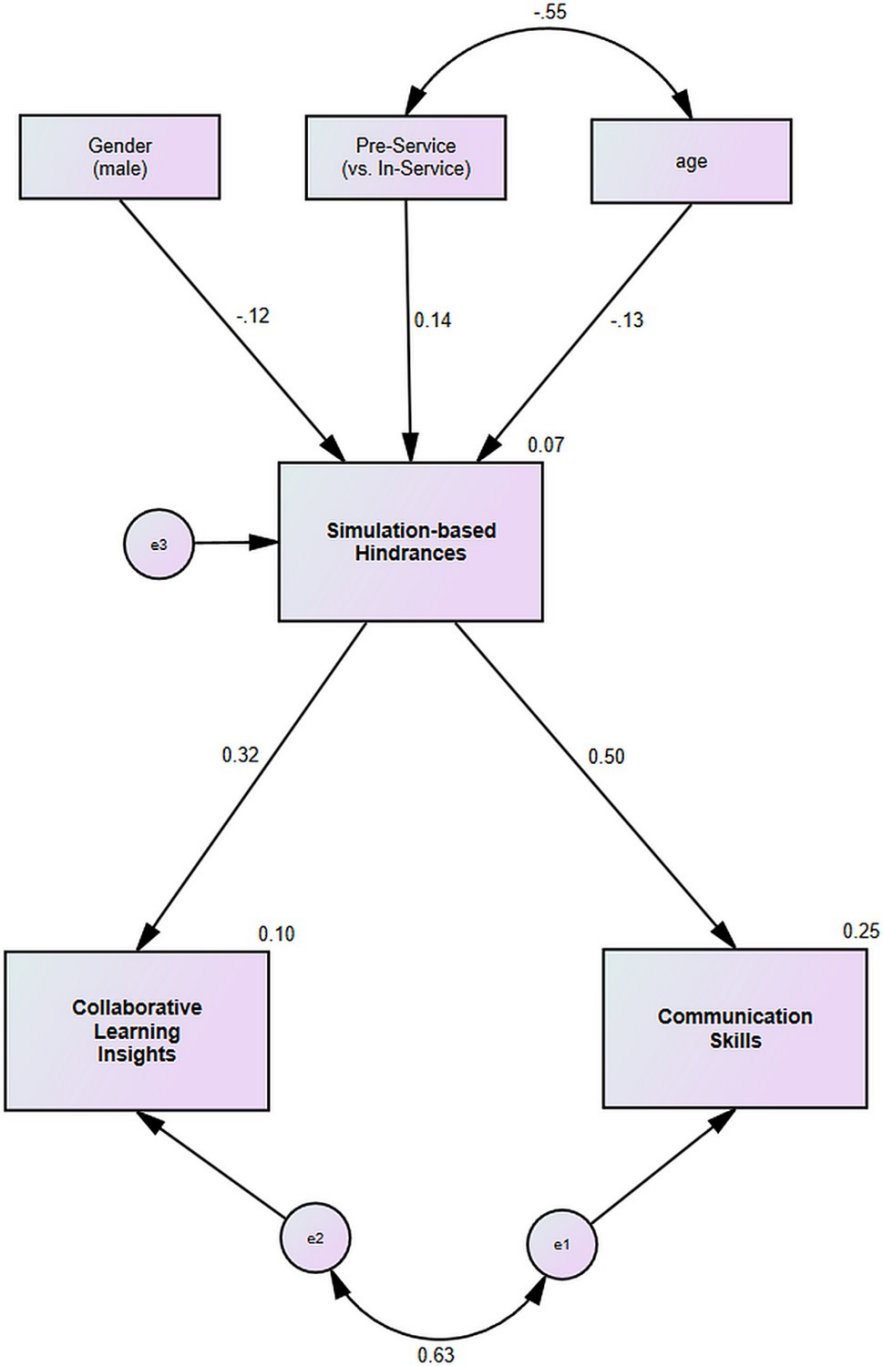
Path analysis for the proposed research model testing the relationships between participants’ communication skills, collaborative learning insights, simulation-related.

The next step in the SEM was to examine the association between the variables (Table 5). Fig 1 shows the standardized effect coefficient (β) of the current study variables and the variability explained (*R*^2^).

**Table 5:** Standardized total effects for the proposed research models testing the relationships between participants’ communication skills, collaborative learning insights, simulation-related hindrances, and background characteristics (*N* = 370).

	Age	Preservice (vs. Inservice)	Gender (male)	Simulation-related Hindrances
Simulation-related Hindrances	-.129	.141	-.115	.000
Collaborative Learning Insights	-.042	.046	-.037	.323
Communication Skills	-.065	.071	-.058	.502

The model revealed a positive association between the hindrance experience and the following two simulation outcomes: communication skills (β = .50) and collaborative learning insights (β = .32), thus supporting research hypothesis (a).

Additionally, the model revealed that the experience of simulation hindrances was lower among older participants (β = -.13), lower among the men than it was among the women (β = -.12), and that it was higher among the preservice teachers than among the inservice teachers (β = .14).

The model also revealed that the simulation-related hindrance variable mediates between all the background variables and the simulation outcomes, as no direct associations were found between the background variables, the participant’s role in the simulation, or the simulation outcomes. The significance of this is that the background variables (gender, age, and professional experience) exerted only an indirect effect on communication skills and collaborative learning insights. These findings support research hypothesis (b).

It is worth noting that no direct significant relationship was found involving either participants’ level of education or the accumulation of professional experience; hence, these variables were excluded from the analysis. This means, that the simulation experience and outcome were not affected by participants’ education level or professional experience.

Furthermore, no significant relationship was found between the participants’ role in the simulation, their simulation-related hindrance, or their learning outcomes (i.e., communication skills or collaborative learning insights). These findings contradict research hypothesis (c), suggesting that participants’ role in the simulation does not significantly affect the SBL experience.

In summary, the results of the analysis show that the background variables are directly related only to the hindrance variable, and the hindrance variable is connected to the two simulation learning outcomes of communication skills and collaborative learning insights. Therefore, the hindrance variable was found to be a mediating variable in SBL.

## Discussion

This study examined the role of simulation hindrances in SBL and found that in the context of teacher education such hindrances mediate the learning. As mentioned previously, the hindrance variable refers to whatever is perceived as potentially hindering the learning process during SBL, such as the experience of stress, inconvenience, embarrassment, etc. The study revealed that experiencing hindrances during SBL mediates the learning outcomes in terms of both the skills learned and the insights gained about collaborative learning. This finding suggests that experiencing hindrances during SBL has a positive facilitating role rather than an impeding one. Thus, the study highlights that unlike traditional arguments for reducing the stress levels during simulations [39, 58], experiencing such hindrances is, in fact, significant to promote optimal learning. This corresponds to the wider ongoing conversation of moving out of one’s comfort zone to enable learning [10] in the field of medical simulations [30], underscoring that this is applicable also in the context of SBL in teacher education.

Given that empirical research on learning through simulations in teacher education is in its early stages, our study enhances theoretical understanding of how learning through simulation occurs and how to best design and implement SBL in this field, with a focus on the potential of these hindrances to learning. This, in turn, may enhance the authenticity of instructional approaches on preservice analysis of their practice [59].

While medical simulation scholars advocated for achieving balance of stress levels to avoid damage [42], our study emphasizes that the stress should not only be "eliminated" or "controlled" but should be deliberately present as it is an essential mediator of the learning outcomes.

Specifically, it was found that women (as opposed to men), preservice (as compared to in-service teachers) and younger learners experienced more hindrances during the simulation; and ultimately these were positively correlated to the learning outcomes.

As regards the teaching status, the study highlights that hindrances during simulations are not only related to personal characteristics [43] but also diverge according to one’s teaching experience. This finding is in line with a recent study on immersive simulations [60], and might be explained by the fact that the in-service teachers have probably had the chance to gain more cumulative experience with conflictual situations; thus, feel less triggered by the simulation. As such, in order to challenge them to leave their comfort zone–to ensure learning–it is recommended to increase the conflict level as well as to provide scenarios that engage in new triggering topics relevant for experienced teachers. Of note, the findings from a previous study, which found that the less central, cognitive and emotion-arousing the conflict, and the higher its level of resolution, the stronger the professional identity formation among the learners [61], raises the need to further examine the level of conflict and its effect on the simulation participants. In this vein, the current findings align with the concept of instructional support (e.g., scaffolding), which should be further explored [62, 63].

As regards gender, prior studies advocate for the need to adjust simulations to the learner’s gender as it was argued that certain aspects of the learning experience are affected by the learner’s gender. Thus, gender could exert an effect on the way the learner in the context of SBL experiences the simulated conflict, the group conversation and its efficacy, and the potential simulation-related hindrances [64]. This difference is important given the majority of women in the field of education in Israel [65], and the professional image of teaching as more “feminine.” In practical terms, this finding indicates the need to take into account the participants’ gender when designing a simulation for SBL.

### Limitations and directions for future research

This study has several limitations. First, this is a cross-sectional study; as such, it does not allow for fully establishing causality. Therefore, the role of hindrances as a learning mediator should be further examined in future studies, particularly in the framework of long-term and longitudinal research designs, as well as in the context of multiple SBL experiences. Second, the sample was predominantly female (92%), which may limit the generalizability of the findings across genders. However, this distribution accurately reflects the gender composition of teachers in Israel, enhancing the relevance of the findings to this specific population. Third, the measure used, i.e., self-reported questionnaires, may be subject to social desirability bias. Further in-depth exploration of the hindrances experience, accompanied by observations, qualitative inquiry, and physiological objective assessments (e.g. heartrate variability (HRV), and Galvanic Skin Response (GSR)) may be useful to better assess the hindrances during simulations and their roles. Future research could also explore the qualitative aspects of the hindrances experienced and how they relate to specific learning outcomes.

Future research may focus on examining the role of simulation hindrances in online simulations, which became prevalent during the COVID-19 pandemic. That is because the element of stress and embarrassment may be experienced differently during an online simulation. Furthermore, future studies should explore the role of cumulative experience in simulation and its relation to the hindrances experience as we found that prior experience with simulation may play a role. Another suggestion for future research should include participants from diverse backgrounds, as people’s sociocultural and religious backgrounds have been found to be essential factors affecting their internal processes [9, 66].

In conclusion, this study illuminates the mediating role of experiencing hindrances for gaining skills and insights in learning through simulations. As such, it contributes to the ongoing conversation regarding the element of emotional stress experienced in simulations and the ways in which learning through simulations can be understood and facilitated to promote teachers’ learning.

## Supporting information

S1 Appendix The Simulation-based Learning Outcomes in Teacher Education (SLOTE) scale–Validated version. (DOCX)
