# Supplementary material for: "No pain, no gain": Simulation-based learning in teacher education: The mediating role of simulation hindrances
Source: PLoS One. 2025 Jan 13;20(1):e0317255. doi: 10.1371/journal.pone.0317255 (PMC11730392; doi:10.1371/journal.pone.0317255)
Supplement: S1 Appendix — (DOCX) [file pone.0317255.s001.docx]

**Appendix 1.**

The Simulation-based Learning Outcomes in Teacher Education (SLOTE) Scale – Validated Version

|  | *Following your participation in the SBL workshop, please indicate on a scale from 1-7 (1 =* not at all; 7 = very much), *the extent to which you agree with the following statements* |
| --- | --- |
| 1 | I learned that it is important to show tolerance and containment. |
| 2 | I've learned how important it is to imagine myself in someone else’s shoes and attempt to understand what they are going through. |
| 3 | I learned to appreciate my colleagues and peers. |
| 4 | I realized that many educators experience difficulties similar to mine. |
| 5 | I have a better understanding of the importance of reflection (self-observation). |
| 6 | I realized that being the object of a peer’s criticism makes it more difficult to learn from the workshop experience. |
| 7 | I now have the tools to deal with situations that I had previously found paralyzing. |
| 8 | Participating in the workshop undermined my belief in my ability to adequately handle professional conflicts. |
| 9 | The workshop allows for the re-examination of real-life professional situations that teachers encounter daily, challenges which we need to think about in depth, but might not have found the opportunity to do so. |
| 10 | I've learned how important it is to choose the right words to convey a message. |
| 11 | The workshop gave me tools for dealing with conflict with authority figures (managers, supervisors, etc.). |
| 12 | I realized that it was important to remain calm- and not respond impulsively. |
| 13 | I learned how certain behaviors can hinder communication. |
| 14 | I've learned how significant body language is (e.g. posture, facial expressions). |
| 15 | The workshop may involve feelings of embarrassment and / or personal exposure that could hinder the learning process. |
| 16 | I realized that keeping my feelings and thoughts pent up inside me simply isn’t worthwhile or effective. |
| 17 | I learned how to be assertive and convey a clear message. |
| 18 | I realized the importance of not letting my ego interfere in my words and actions. |
| 19 | I learned how I should start a conversation. |
| 20 | I've learned which of my behaviors I value and want to keep. |
| 21 | I've learned what I need to improve in my behavior. |
| 22 | I have gained something that may be beneficial also in my personal life. |
| 23 | I learned how to express empathy towards the people with whom I interact. |
| 24 | I acquired tools to deal with situations of conflict with others. |
| 25 | I've learned to withhold judgement and to listen to the other’s point of view. |
| 26 | I learned the importance of asking open-ended questions. |
| 27 | I realized that when I face any situation there are many details and aspects that I am not sufficiently aware of. |
| 28 | I realized that the problems that arise should be approached professionally, rather than personally. |
| 29 | Following the workshop, I have had second thoughts about whether to continue in this career path or even in this professional field. |
